# Association of High Cardiovascular Fitness and the Rate of Adaptation to Heat Stress

**DOI:** 10.1155/2018/1685368

**Published:** 2018-02-28

**Authors:** Małgorzata Żychowska, Alicja Nowak-Zaleska, Grzegorz Chruściński, Ryszard Zaleski, Jan Mieszkowski, Bartłomiej Niespodziński, Roman Tymański, Andrzej Kochanowicz

**Affiliations:** ^1^Department of Life Sciences, Faculty of Physical Education, Gdansk University of Physical Education and Sport, Gdańsk, Poland; ^2^Plasmids and Microorganisms Collection, Faculty of Biology, University of Gdańsk, Gdańsk, Poland; ^3^Department of Management in Sport, Faculty of Physical Education, Gdansk University of Physical Education and Sport, Gdańsk, Poland; ^4^Non-Public Health Centre “ETERMED”, Gdańsk, Poland; ^5^Department of Anatomy and Biomechanics, Institute of Physical Education, Kazimierz Wielki University, Bydgoszcz, Poland; ^6^Department of Sport, Gdansk University of Physical Education and Sport, Gdańsk, Poland; ^7^Department of Gymnastics and Dance, Gdansk University of Physical Education and Sport, Gdańsk, Poland

## Abstract

This study aimed to compare changes in genes expression associated with inflammation and apoptosis in response to heat stress caused by sauna between people with varying cardiorespiratory fitness levels. We hypothesis that high cardiorespiratory level caused higher positive changes after four weeks of sauna bathing. Blood samples were taken at rest before and after the first and last sauna sessions and 48 hours after the last sauna session and used to assay HSP70* (HSPA1A)*, HSP27* (HSPB1)*, interleukin 6* (IL6)*, and interleukin 10* (IL10)* genes expression in blood with quantitative real-time qRT-PCR. Overall, small decreases in rest values of* HSPA1A* and* IL6 *mRNA, increase in* HSPB1* mRNA, and a significant increase in* IL10* mRNA were observed after four weeks of exposure to heat stress. Our findings suggest that an adaptive response to heat stress (an anti-inflammatory response) occurs faster in people with higher cardiorespiratory fitness.

## 1. Introduction

Finnish sauna bathing is a very popular and easily accessible wellness treatment in sports, recreation, and rehabilitation. The effects of sauna bathing can be positive or negative, depending on many factors, for example, differences in physical condition, disease status, and external conditions. Sauna bathing has been observed to influence skeletal muscle [1], reduce oxidative stress [2], activate secretion of adrenocorticotropic hormone (ACTH) and cortisol in young women [3], stimulate the immune system [4], affect genes expression [5, 6], and impact the functionality of other organs (e.g., reducing the viability of sperm cells) [7].

The physiological systems most impacted by sauna sessions are the skin and the thermoregulatory and cardiovascular systems [8]. Despite a normal strong response of the thermoregulatory system, involving an increased production of sweat, internal body temperature typically increases during a dry sauna. Overheating and dehydration are stressors that activate homeostatic mechanisms, such as the increased expression of stress-related genes at the molecular level. Stress-related genes can be overexpressed, especially those associated with apoptosis and the degradation of damaged and denatured proteins, such as Hsp70* (HSPA1A)* or Hsp27* (HSPB1) *[9, 10]. These two genes are commonly induced by physiological stress [11–14]. Recently, in the literature there are some studies in which impact of sauna bathing on the human body was investigated in active and sedentary people on transcriptional level [5, 6]. Cited studies showed that* IL6* mRNA and* CRP* mRNA were higher in people with lower cardiorespiratory fitness after single sauna session [5]. Moreover, sauna can cause higher changes in genes expression (*HSPA1A*,* HSPB1*,* IL6*, or* IL10* mRNA) than moderate exercise [6].

Repeated thermal stress can induce thermos tolerance at the cellular level and that is important for health and physiological possibilities and translates into efficiency in work performed at high temperatures. So we investigated the adaptive effect of four weeks of sauna bathing on the expression of the genes* HSPA1A*,* HSPB1*,* IL6*, and* IL10*. Expression of the genes coding for heat shock proteins (*HSPA1A* and* HSPB1*) and interleukins (*IL6* and* IL10*) is particularly interesting in sports medicine because these genes appear to have the effect of increasing exercise tolerance [6]. We hypothesized that the adaptive effect of total body exposure to the heat stress associated with sauna sessions would vary as a function of the physical condition of the experimental subjects. Specifically, we hypothesized that the sauna-induced increases in the expression of the genes mentioned above would be higher in nonathletes than athletes and any adaptive effect would appear earlier because of the cellular thermotolerance athletes acquire during physical training. Then people with higher cardiorespiratory system easier tolerate work in high temperatures.

As the type of training may affect gene expression [15], we chose to study athletes playing the same sport. We therefore monitored footballers, who we considered the experimental group, and sedentary people, who were considered to be the control group. In our opinion, differences in responses between groups for sauna bathing may be similar to those obtained by physical exertion [6]. Therefore we hypothesized that the upregulation of anti-inflammatory rather than proinflammatory interleukins would be faster in people with higher cardiorespiratory fitness.

## 2. Material and Methods

### 2.1. Ethics Statement

The study was approved by the Bioethics Committee for Clinical Research at the Regional Medical Chamber in Gdańsk (KB14/14). The authors were obliged to respect the principles of the Helsinki Declaration. All participants gave written, informed consent to participate in the study and could withdraw consent at any time for any reason.

### 2.2. Participants

Twenty-two healthy men participated in the experiment: eleven athletes playing football (average career length was 8 years) and eleven nonathletes. No significant differences between the groups' body mass, BMI, and body height were found (Table 1).

Athletes trained for 2 sessions per day, 6 days per week (25–28 hours). Control group participants reported recreational physical activity such as running, swimming, or team sports maximum 2 times a week, for a duration of 45 minutes per session. All subjects had a normal health status during the two months prior to the study (no drugs, alcohol, nicotine, negative medical history for injures, and situations that may influence the results).

### 2.3. Study Design

Participants avoided use of a sauna and intensive sunbathing for a month before the experiment and during the time of experiment (beyond the impact of the experiment). The experiment was performed in January during a break in training and competitive football for the athletes; participants in the control group reported average levels of spontaneous physical activity during the study period (on average 2 sessions per week). Athletes and the control group participated in a dry sauna bath 3 times a week, for 4 weeks. Participants spent time in a Finnish sauna room at 98.2°C and 10 ± 2% humidity; the time was comprised of two 15-minute stints during the same session (total time: 30 min per session) with a 5-minute break for cooling under the shower (water temperature was 18 ± 2°C). Before and after each session in the sauna, weight and percent body fat were measured using an InBody 720 Segmental Body Composition Analyser, Biospace (Korea).

### 2.4. RNA Extraction, qRT–PCR

Two millimeters of venous blood was collected five times: sample (1) was taken immediately before the first sauna, sample (2) 15 minutes after the first sauna, sample (3) immediately before the twelfth sauna, sample (4) 15 minutes after the twelfth sauna, and sample (5) 48 hours after the twelfth sauna. The samples were processed immediately after each collection. Erythrocytes were lysed using RBCL buffer according to the manufacturer's instructions (A&A Biotechnology, Gdynia, Poland). The remaining white blood cells were lysed using Fenozol according to the manufacturer's instructions (A&A Biotechnology, Gdynia, Poland). Isolation of total RNA was carried out by a chemical method described by Chomczynski and Sacchi [16]. Purity and concentration of the isolated RNA were determined by spectrophotometry (Eppendorf, BioPhotometer Plus, Germany). cDNA synthesis from 2 micrograms of total RNA was performed with a TranscriptMe Kit, using oligo dT and random hexamers (Blirt, Gdańsk, Poland).

### 2.5. Quantitative Polymerase Chain Reaction (q-PCR) Assay to Determine Gene Expression

For the analysis of gene expression, real-time PCR (LightCycler 480II, Roche, Poland) was twice performed in triplicate for each sample using LightCycler polymerase (Roche, Poland). The temperature-time protocol of each reaction was programmed according to the manufacturer's instructions. For each reaction, melting curve analysis was performed. The B-tubulin* (TUBB)* was used as a reference gene. Primer sequences are shown in Table 2.

### 2.6. Statistical Analysis

The data were collected and relative gene expressions were analysed in Excel 2005. In order to calculate the level of gene expression, the comparative *C*_*T*_ method of Schmittgen and Livak [17] was used. The results are expressed as mean *C*_*T*_ values and standard deviations. The data were transformed to linear values and to assess statistical significance, the following tests were used: normal distribution was checked with the Shapiro-Wilk's test, and the nonparametric Wilcoxon test was used to compare results before and after the sauna baths. To determine the significance of differences between the groups, a one way ANOVA was applied. All calculations and graphics were performed using GraphPad Prism 6.0 (ftx.pl/program/graphpad-prism). Statistically significant differences were considered at a level of *p* ≤ 0.05.

## 3. Results

### 3.1. Changes in Somatic Indicators and Dehydration

Small changes of body weight and percent body fat were observed before and after four weeks of sauna sessions in both groups: the mean weight of the group of athletes decreased from 72.26 ± 2.4 kg to 71.78 ± 3.8 kg (a decline of 0.48 kg), while the mean weight of the nonathletes decreased from 77.6 ± 5.7 to 77.3 ± 5.5 kg (a decline of 0.3 kg). Weight change due to dehydration during a single session in the sauna ranged from 0.42 to 1.1 kg. There were no significant differences either within or between groups in terms of loss of body mass, dehydration, or duration of their stay in the sauna.

### 3.2. Genes Expression

**Figure 1:**
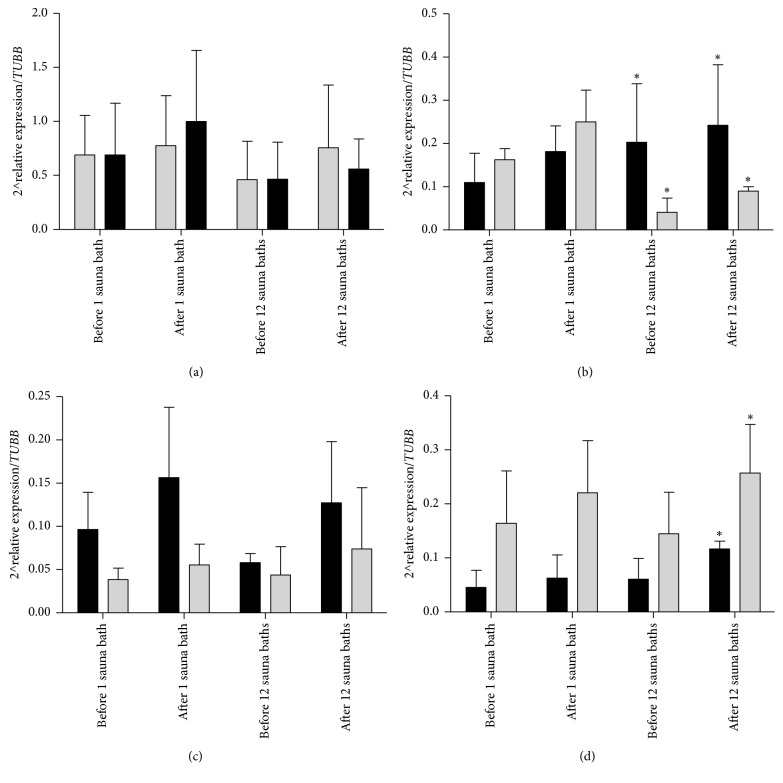
2^∧^relative expression in athletes (gray bars) and control group (dark bars) before and after first and last sauna bathing. (a)* HSPA1A*, (b)* HSPB1*, (c)* IL6*, and (d)* IL10*. ^*∗*^*Significant differences between groups* (*p* < 0.05).

Rest value of* HSPA1A* mRNA was slightly higher in sedentary people before saunas (first and last) and after first sauna bathing, contrary to* HSPB1* mRNA (higher before and after first sauna bathing and significantly lower before and after last sauna bathing, Figures 1(a) and 1(b)). Changes in relative expression of* HSPB1* mRNA were from 2^∧^0.2 before last sauna to 2^∧^0.24 after last sauna in control group and 2^∧^0.04 before last sauna to 2^∧^0.08 after last sauna in athletes (*p* = 0.004). Changes in* IL6* mRNA caused by first and last sauna bathing were lower in athletes while in* IL10* mRNA they were significantly higher at the same time, significantly after last sauna bathing (2^∧^0.11 in control and 2^∧^0.25 in athletes, *p* = 0.04, Figures 1(c) and 1(d)).

**Figure 2:**
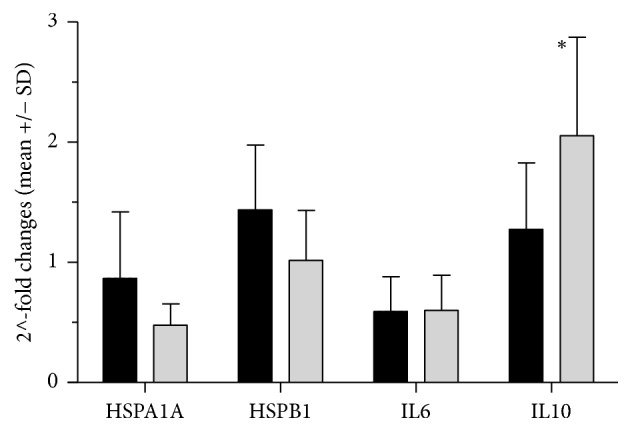
2^∧^-fold changes in the expression between rest before and 48 h after experiment in athletes (gray bars) and sedentary people (dark bars). 2^∧^-fold changes were calculated as 2^∧^-fold changes before/2^∧^-fold changes after experiment. ^*∗*^Significant changes between rest value before experiment and 48 h after experiment (*p* ≤ 0,05).

Adaptation to heat stress was determined by comparison rest value before first sauna bathing and 48 h after last sauna bathing and results as 2^∧^-fold changes are presented at Figure 2.

**Figure 3:**
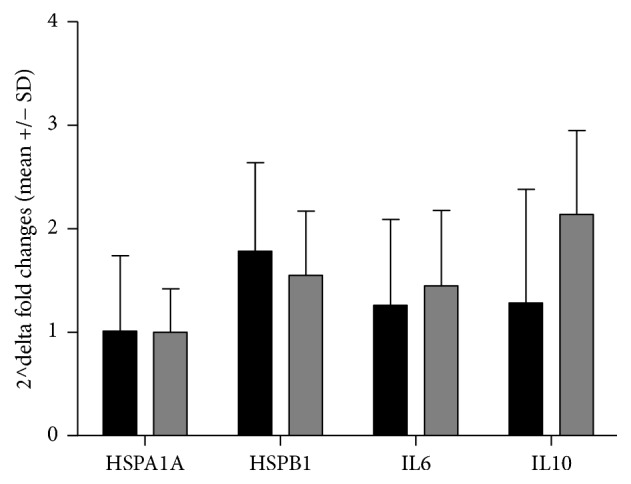
Differences in 2^∧^-fold changes (delta 2^∧^fold) in response to first and last (12) saunas in athletes (gray bars) and sedentary people (dark bars). Delta 2^∧^-fold changes = 2^∧^-fold changes after last sauna bath (rest value/post rest value)/2^∧^-fold changes after first sauna (value before/after sauna).

Decreases in the expression of* HSPA1A *and* IL6 *mRNA in response to heat stress were observed after four weeks of sauna sessions in both groups, while expressions of* IL10* mRNA in athletes significantly increased in the time span and were 2^∧^2.04-fold in athletes and 2^∧^1.27-fold in sedentary people, *p* = 0.04. For* HSPA1A *mRNA, decreases in 2^∧^-fold changes were slightly higher in athletes (to 2^∧^0.6-fold in athletes and to 2^∧^0.85-fold in sedentary people). For all genes tested, similar adaptive effects were reported for each individual group. The differences in response in genes expression obtained after first and last (12) sauna sessions are presented at Figure 3.

No changes in delta 2^∧^-fold changes (2^∧^-fold changes after first sauna/2^∧^-fold changes after last sauna) in* HSPA1A* were observed in both groups, while increases in* HSPB1*,* IL6,* and* IL10* mRNA were seen. Changes in response in* IL6* mRNA after the last sauna compared to the first one differed between groups and were 2^∧^1.44-fold in athletes and 2^∧^1.26-fold in sedentary people, while in* IL10* mRNA they were 2^∧^2.14-fold in athletes and 2^∧^1.28-fold in sedentary people. The differences between groups in delta 2^∧^*x*-fold changes were not significant (ANOVA one way).

## 4. Discussion

There are a number of studies examining the effect of high temperature on gene expression, but we are not aware of* in vivo* investigations of mRNA levels of stress-related genes after sauna bathing. Furthermore, little has been reported on the impact of physical activity on the molecular response of leukocytes to the same stressor. The small differences in expression seen in this study occurred between a sample taken at rest before and 48 hours after a course of 12 sauna baths. Four weeks of sauna sessions caused a significant increase in* IL10* mRNA in people with high cardiorespiratory fitness. In our studies faster adaptation to heat was observed in active people.

Stress-related genes are overexpressed after exercise, as shown by Neubauer et al. [18], Büttner et al. [19] and Radom-Aizik et al. [20], Rasmus et al. [21], and Szołtysek et al. [22]. Regardless of the stressor, a cellular response involves several hundred stress genes, including production of heat shock proteins or interleukins [23, 24]. Increased expression of genes encoding anti-inflammatory interleukins like* IL10* can result in lower expression of* IL6* mRNA, important indicators of inflammation. Four weeks of sauna bathing caused a decrease in rest value of* IL6* mRNA (not significant) and an increase in* IL10* mRNA (significant in active people).

Stimulation of the synthesis of heat shock proteins and interleukins in leukocytes in response to stressors including temperature was studied by Pizurki and Polla [25] and Jacquier-Sarlin et al. [26]. Using* in vitro* study of leukocytes, these authors found that increasing the temperature to 41°C caused a 10-fold increase in HSPs [25, 26]. The aim of our study was to determine adaptive effect to heat stress, but our results were not this high. This difference confirms the substantial differences between* in vivo* and* in vitro* testing [27]. However, repeated heat stress caused an visible increase in the expression of* HSPB1* mRNA at rest value and after 12 sauna baths compared to the first sauna bath and decrease expression of* HSPA1A* and* IL6 *mRNA at rest value (Figures 2 and 3). By analysing changes in the expression of* HSPB1, IL6*, and* IL10 *mRNA, it can be seen that a four-week course of whole body exposure to high temperature caused slightly higher expression of these genes in response to the last saunas compared to the first one. Changes in rest value showed decrease in* HSPA1A* and* IL6* mRNA and increase in* HSPB1 *and* IL10* mRNA, measured before experiment and 48 hours after last sauna session. The same thermal load caused in the similar direction of changes occurred faster in people with higher cardiorespiratory fitness. It is possible that adaptive effect to heat stress occurs faster in physically active people.

Based on our results, we suggest that the adaptation of regulation of genes associated with apoptosis and the immune response to heat stress may be associated with level of physical activity. Furthermore, the changes achieved in our groups after four weeks of overheating consisting of decrease in proinflammatory and increase in anti-inflammatory genes expression indicated positive impact on health (more pronounced in athletes). This is an important conclusion for planning biological regeneration and for people who work in environment with elevated temperature as it improved work efficiency and acclimatization will occur faster in people with high cardiovascular fitness.

## Figures and Tables

**Table 1:** Anthropometric characteristics of the study groups.

	Age (year)	Height (cm)	BMI	Fat (%)	Training experience (years)
Athletes (mean ± SD)	19.5 ± 0.53	179.87 ± 7.75	22.07 ± 1.94	8.21 ± 2.24	8 ± 1.5
Nonathletes (mean ± SD)	19.67± 0.87	183.78 ± 6.83	22.89 ± 2.33	11.34 ± 4.47	-

**Table 2:** Primers used to real-time PCR.

*HSPA1A*	Forward primer: TGGACTGTTCTTCACTCTTGGC Reverse primer: TTCGGAGAGTTCTGGGATTGTA
*HSPB1*	Forward primer: AAGGATGGCGTGGTGGAGATCA Reverse primer: GAGGAAACTTGGGTGGGGTCCA
*IL6*	Forward primer: TCCACGGCCTTGCTCTTGTTT Reverse primer: GACATCAAGGCGCATGTGAAC
*IL10*	Forward primer: GAATCCAGATTGGAAGCATCC Reverse primer: AATTCGGTACATCCTCGACGG
